# Automatic load sharing of distribution transformer for overload protection

**DOI:** 10.1186/s13104-019-4880-1

**Published:** 2020-01-07

**Authors:** Abraham Hizkiel Nebey

**Affiliations:** Bahir Dar University Institute of Technology, P.O.Box 26, Bahir Dar, Ethiopia

**Keywords:** Transformer overload, Fuzzy logic controller, Dangila, Mat lab/Simulink

## Abstract

**Objective:**

Load sharing provides sufficient protection to distribution transformer under overloaded conditions. Due to overload on transformer, the efficiency drops and windings get overheated and may burn. By sharing a load current on transformer for each phase the transformer was protected. Therefore, the objective of this study was to protect transformers from overloaded conditions by sharing the load.

**Result:**

The system automatically connects and disconnects switch to share the transformer loads. The controller was managed the load according to rules.

## Introduction

Overloading protection means detecting problems with distribution transformer and isolating from the load. Fuzzy logic controller is an intelligent tool that serves as an overloading protection for distribution transformers. It has logical rules which protects distribution transformers against overloading conditions [1].

Overloading protection for the distribution transformer can reduce around 20% of electric power interruption [2]. Protection and overloading protection in a particular, is one of the measurement strategies to improve power system’s reliability status.

In Ethiopia, many customers were suffering from electric power interruptions. There were many malfunctions distribution transformers in Dangila town. Major causes of this malfunction of the distribution transformer was overloading conditions which can be easily prevented through overloading protection [3–5]. Currently, the practice of overloading protection in Ethiopia is low [3, 6, 7].

Even though the government of Ethiopia developed strategies for energy supply, there is the varying level of intervention were being given both at the community level and energy centres [8]. The efforts were not organized at the level of practice and this is due low number of researches to explore overloading protection in Ethiopia [9–11]. Therefore, the aim of this study was to protect the distribution transformers in Dangila town, Ethiopia.

## Main text

### Methods

#### Study setting and period

A Failure Modes and Effects Analysis were conducted in Dangila town from April 1 to June 3, 2019. Dangila is the capital city of Dangila woreda in Amhara regional state. It is 78 km away from Bahir Dar which is the capital city of Amhara regional state. The city has five sub towns, one substation, which gives serves to the town and the surrounding people.

Questionnaires were distributed to Ethiopia Electric Utility maintenance staffs. The questionnaire assesses the consequences of failures in terms of electric power interruption, damage of the components or further damage of subsequent sub systems.

### Measurement

Data were collected using interview and direct measurement. The questionnaire was prepared to collect the relevant data [3, 12]. Two weeks were given for data collectors and supervisors for 3 days on methods of extracting the information through interviewing, and direct measurement.

#### Mathematical modelling for determining KVA ratings

There were a mathematical equation for determining KVA rating of transformer, one and two.

From the collected data following parameters were calculated.

**For load assessment area of A**

Real power

$$\begin{aligned} {\text{P}} & = \left( { 1 7 50 20 + 1 4 700 + 3 4000 + 40 50} \right)\,{\text{W}} \\ & = 2 2 7 7 70\,{\text{W}} \\ & = 2 2 7. 7 70\,{\text{kW}} \\ \end{aligned} .$$

From this the load current can be calculated

1 $$\begin{aligned} {\text{I}}_{\text{L}} & = \frac{\text{real power}}{\sqrt 3*Vs *pf} \\ & = \frac{{227770\,{\text{W}}}}{1.73 *380 *0.8} = \frac{{227770\,{\text{W}}}}{{526\,{\text{V}}}} = 433\,{\text{A}} \\ \end{aligned} .$$

Then to find the KVA rating of the transformer A,

2 $$\begin{aligned} {\text{KVA}}_{\text{A}} & = \frac{\text{real power}}{PF} \\ & = \frac{227.770}{0.8}\,{\text{kW}} = 2 8 5\,{\text{KVA}} .\\ \end{aligned} .$$

From this the transformer rating was selected. For safety purposes and expansion, in case more loads were added at a later date. Therefore, transformer rating was approximately 315 KVA.

**For load assessment area of B**

In similar way:

Rated power were the sum of the entire total load that delivered to the consumer.

$$\begin{aligned} {\text{Real power}} & = \left( { 1 8 3 40 + 1 8 7 2 8 5+ 3 4000} \right)\,{\text{W}} \\ & = 2 3 9 6 2 5\,{\text{W}} \\ & = 2 3. 9 6 2 5\,{\text{KW}} \\ \end{aligned}.$$

From these load currents was calculated as;

3 $$\begin{aligned} {\text{I}}_{\text{L}} & = \frac{\text{real power}}{{\surd 3 * {\text{vs}}*{\rm pf}}} \\ & = \frac{{23.9625\,{\text{KW}}}}{{1.73 *380{\text{v}}*0.8\,{\text{V}}}} = \frac{{23.9625\,{\text{KW}}}}{{526\,{\text{V}}}} = 4 5 5. 5\,{\text{A}} .\\ \end{aligned} .$$

Then to find the KVA rating of the transformer B

4 $$\begin{aligned} {\text{KVA}}_{\text{B}} & = \frac{\text{Real power}}{PF} \\ & = \frac{{242.245 \,{\text{KW}}}}{0.8} = \; 30 3\;{\text{KVA}} .\\ \end{aligned}$$

From this the transformer rating was selected above this value because the transformer rating was greater than the actual load. This was done for safety purposes and expansion, in case more loads were added at a later date. Therefore, the transformer rating was approximately 315 KVA.

#### Mathematical modelling of two load sharing of transformers

Two transformers of equal voltage ratios were selected for working in parallel. This can avoid a circulating current between the transformers [13–16].

**Fig. 1:**
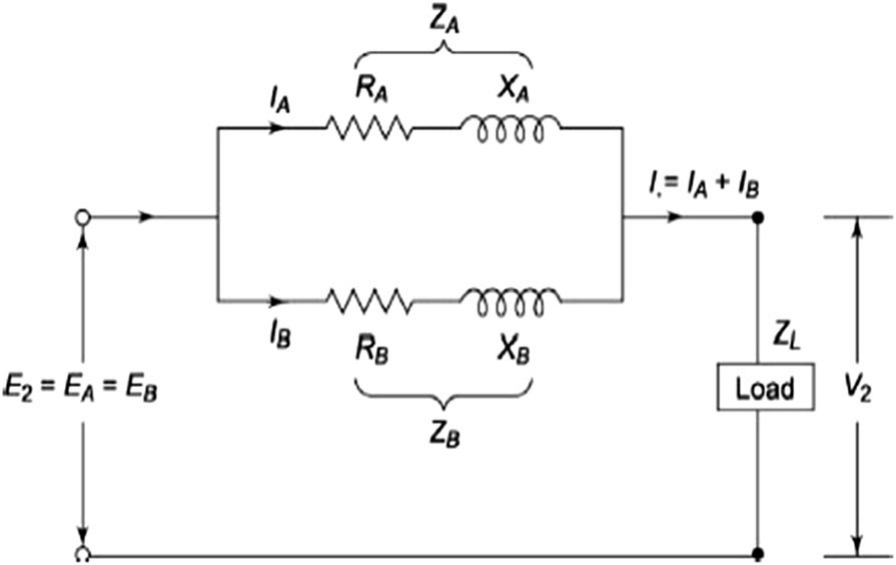
Equivalent circuits of parallel transformers

From the above (Fig. 1) circuit the following parameters were calculated.

5 $${{\text{I}}_{\text{A}}} {{\text{Z}}_{\text{A}}} = {{\text{ I}}_{\text{B}}}{ {\text{Z}}_{\text{B}}} = {{\text{ I}}_{\text{L}} }{{\text{Z}}_{\text{L}} }= {\text{ V}}\left( {\text{say}} \right)$$

6 $${\text{Z}}_{\text{A}} = {\text{R}}_{\text{A}} + {\text{X}}_{\text{A}}$$

7 $${\text{Z}}_{\text{B}} = {\text{R}}_{\text{B}} + {\text{X}}_{\text{B}}$$

Here,

8 $${\text{I}}_{\text{L}} = {\text{I}}_{\text{A}} + {\text{I}}_{\text{B}}$$

And Z_equ_ is the equivalent impedance of the two transformers given by,

9 $${\text{Z}}_{\text{equ}} = \frac{{{{\text{Z}}_{\text{A}} }{{\text{Z}}_{\text{B}} }}}{{{\text{Z}}_{\text{A}} + {\text{Z}}_{\text{B}} }}$$

From Eqs. (5), (6) and (7)

10 $${\text{I}}_{\text{A}} = \frac{V}{{Z_{\text{A}} }} = \frac{{I_{\text{L}} Z_{\text{L}} }}{{Z_{\text{A}} }} = I_{\text{L}} \frac{{Z_{\text{B}} }}{{Z_{\text{A}} + Z_{\text{B}} }}$$

11 $${\text{I}}_{\text{B}} = \frac{V}{{Z_{\text{B}} }} = \frac{{I_{\text{L}} Z_{\text{L}} }}{{Z_{\text{B}} }} = I_{\text{L}} \frac{{Z_{\text{A}} }}{{Z_{\text{B}} + Z_{\text{A}} }}$$

From the above it has seen that the transformer with higher impedance supplies lesser load current and vice versa. If transformers of dissimilar ratings were paralleled the transformer with a larger rating shall have a smaller impedance as it has to produce the same drop as the other transformer, on a larger current. Thus the ohmic values of the impedances must be in the inverse ratio of the ratings of the transformers.

$${{\text{I}}_{\text{A}}}{ {\text{Z}}_{\text{A}}} = {{\text{ I}}_{\text{B}}}{ {\text{Z}}_{\text{B}}} ,\;{\text{therefore}} \ \frac{{{\text{I}}_{\text{A}} }}{{ {\text{I}}_{\text{B}} }} = \frac{{ {\text{Z}}_{\text{B}} }}{{Z_{\text{A}} }}$$

### Modelling of fuzzy logic base protective relay

The Fuzzy logic interface (Additional file 1: Figure S1) was used to make decisions [17–19]. Thus it is more precise than conventional relaying techniques. The Fuzzy logic controller was an intelligent tool to manage the loading condition, so as to protect transformers from overload conditions.

The proposed fuzzy logic based input was defined as:

- T_1L,_ represents the load of transformer one.
- T_2L,_ represents the load of transformer two.

#### Membership function for proposed control

The membership function defined how the input is mapped to member values [20–23].

- **T**_**1L,**_ represents the membership function load of transformer one (Additional file 2: Figure S2). It consists small, medium and large.
- **T**_**2L,**_ represents the membership function load of transformer Two (Additional file 3: Figure S3). It consists small, medium and large.

The output power was membership functions of the proposed control system (Additional file 4: Figure S4). The output linguistic values were not shared, share, and trip (Table 1).

**Table 1:** Rule base of transformer load

T_2L_	T_1L_		
	Small	Medium	Large
Small	Not share	Not share	Share
Medium	Not share	Not share	Share
Large	Share	Share	Trip

#### Proposed control rule

**Fig. 2:**
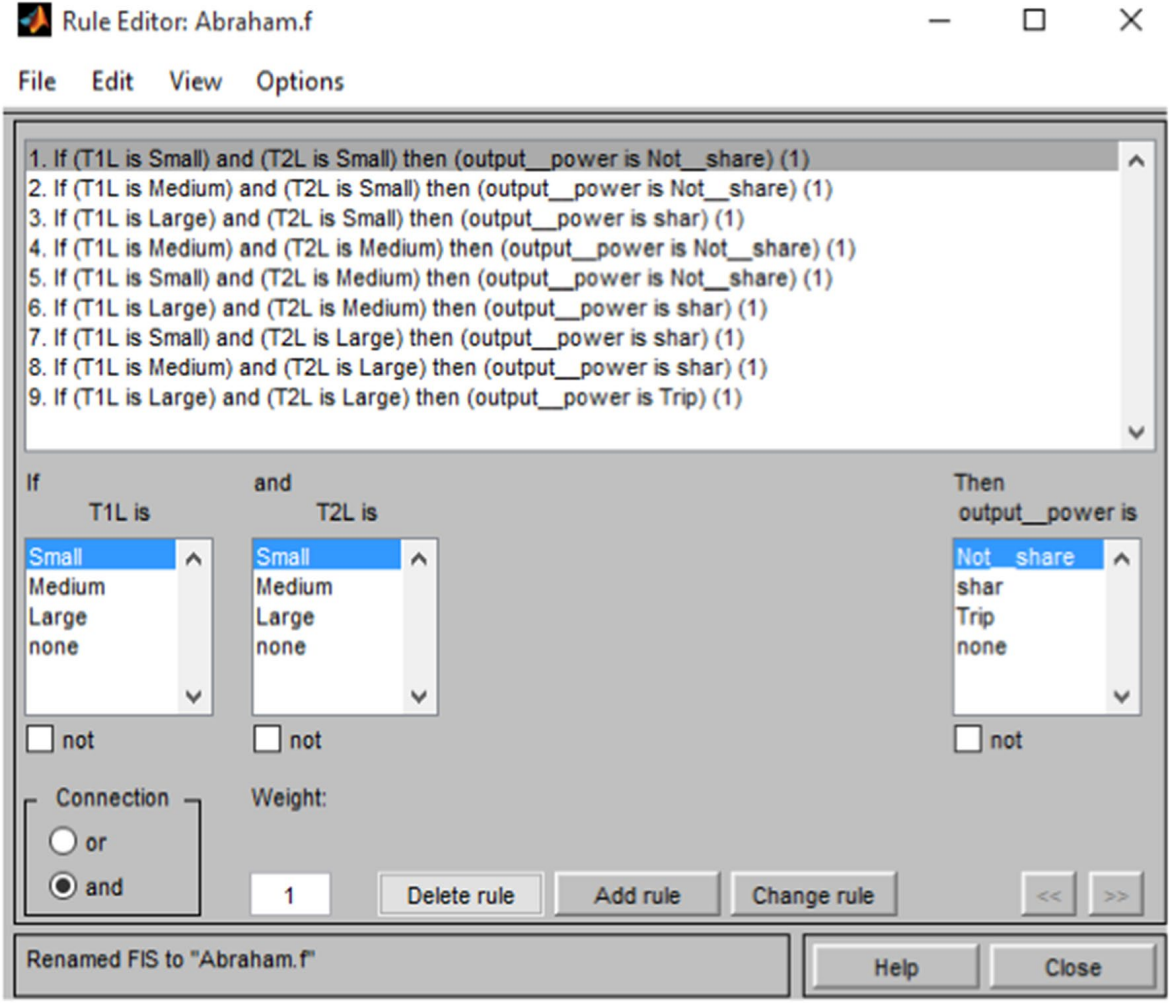
Rule editor

The control rule content was linguistic variables [24–27]. These linguistic descriptions take from if then rule. The proposed control had nine rules for transformer overloading protection that consist input antecedents and consequences of output (Additional file 5: Figure S5). The rules are shown in (Fig. 2).

#### Overall controller system

Transformer load one and transformer load two were the input parameters of the controller. T_1L_ and T_2L_ indicates the load of transformer one and load of transformer two respectively. The load for each transformer was assumed to the Gaussian random signal generator. The multiport conditional switch (Additional file 6: Figure S6) will take an action according to the rules written and loaded in the fuzzy logic controller.

#### Operational definition

Overloading protection: If power system protection were used to protect the distribution transformer from overloading conditions [20, 28–31].

## Result and discussion

### Simulation result

The controller looks the load on transformer first and made decisions (not share, share and trip) to protect transformers. Therefore, there was no equipment failure and power interruption due overloading conditions.

In order to evaluate the performance of rule based load sharing system, three various types of loads were applied at the output of the distribution transformer

- Case (I): The two transformers were at normal state.
- When the two transformers were at normal state, during this time the sharing relay was not energized because it feeds its load at normal state.
- Case (II): when one of the transformers was overloaded.
- When one transformer was at normal state and another was overloaded, at this condition the sharing relay was energized to share the load.
- Case (III): The two transformers were overloaded for each phase.

When two transformers were overloaded, at this condition the relay will trip to protect transformers.

## Conclusion

The work was all about how to supply power intelligently manage an overload condition. The system automatically connects and disconnects switch to share the transformer loads. Most of the villages were suffering with electric instructions. This was due to transformer failure. A recommendation to decrease power interruption in the town was: Load sharing of transformer to protect from failures. This load sharing was modelled to the town; therefore, this transformer load sharing system was the best solution to protect equipment failure and electric instructions.

Intelligent controller was used to make intelligent decisions by sensing amount of the load on the transformer. Rule base fuzzy logic controller manages the load and transformers were protected from overload condition.

### Limitations

1. Possible demand growth rate with population and economic growth rate (load forecasting).
2. Further study can be carried out for sensing fault current, surge voltage (consider more parameters).

## Supplementary information

**Additional file 1: Figure S1.** Fuzzy logic interface model.

**Additional file 2: Figure S2.** Membership functions of transformer load one.

**Additional file 3: Figure S3.** Membership functions of transformer load two.

**Additional file 4: Figure S4.** Membership functions of output power.

**Additional file 5: Figure S5.** Rule evaluations.

**Additional file 6: Figure S6.** Overall protection systems for overloading protection/interface.

## Data Availability

The date of this study will not be shared publically due to the presence of sensitive (confidential) participants’ information.

## Abbreviations

KVA: kilo volt ampere
KWh: kilo Watt hour
P: real power
