# Supplementary figures and images for: Automatic load sharing of distribution transformer for overload protection
Source: BMC Res Notes. 2020 Jan 7;13:17. doi: 10.1186/s13104-019-4880-1 (PMC6947968; doi:10.1186/s13104-019-4880-1)

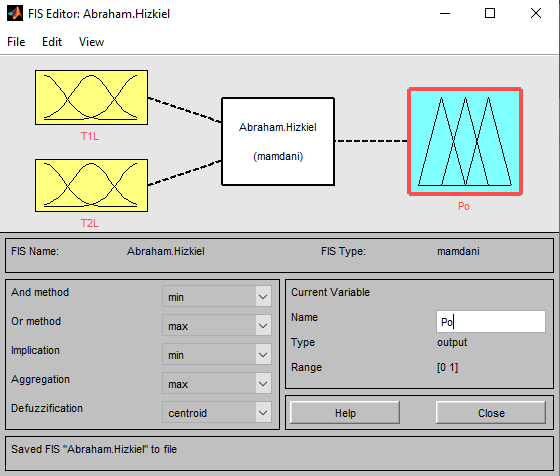


Figure S1. Fuzzy logic interface model

Supplement: Supplementary file 1 — Additional file 1: Figure S1. Fuzzy logic interface model. [file 13104_2019_4880_MOESM1_ESM.docx]

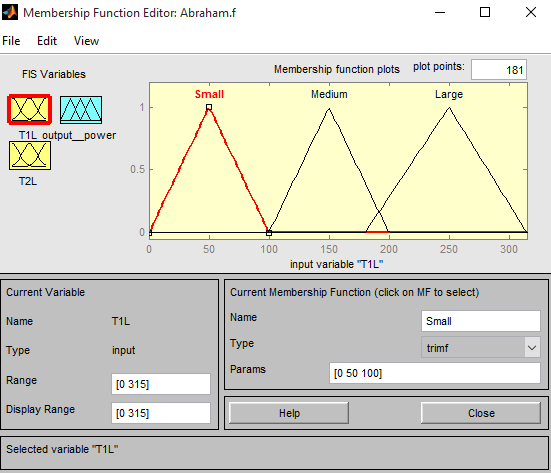


Figure S2. Membership functions of transformer load one

Supplement: Supplementary file 2 — Additional file 2: Figure S2. Membership functions of transformer load one. [file 13104_2019_4880_MOESM2_ESM.docx]

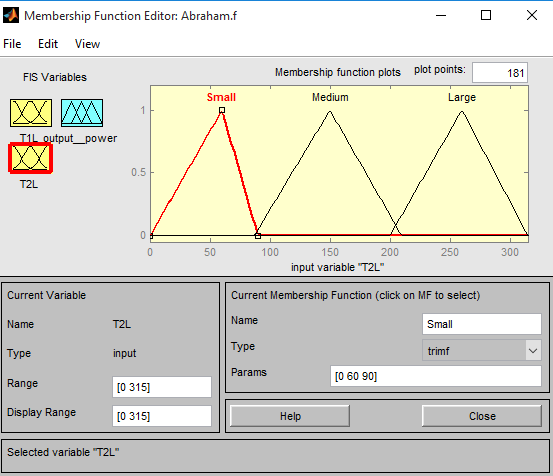


Figure S3. Membership functions of transformer load two

Supplement: Supplementary file 3 — Additional file 3: Figure S3. Membership functions of transformer load two. [file 13104_2019_4880_MOESM3_ESM.docx]

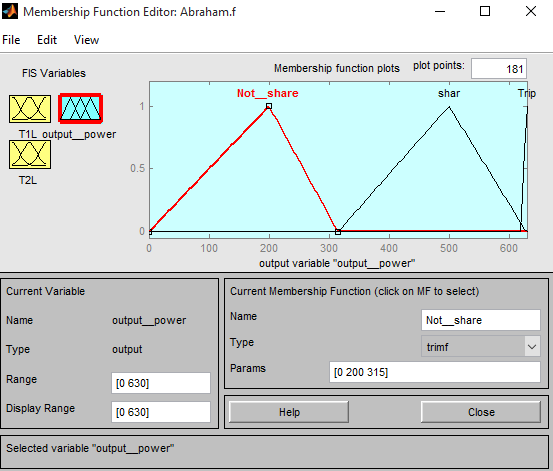


Figure S4 Membership functions of output power

Supplement: Supplementary file 4 — Additional file 4: Figure S4. Membership functions of output power. [file 13104_2019_4880_MOESM4_ESM.docx]

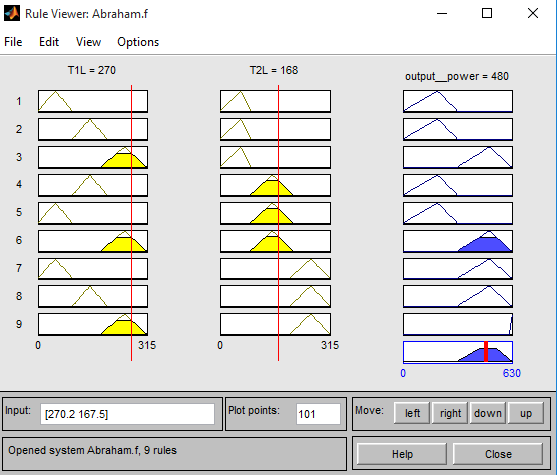


Figure S5. Rule evaluations

Supplement: Supplementary file 5 — Additional file 5: Figure S5. Rule evaluations. [file 13104_2019_4880_MOESM5_ESM.docx]

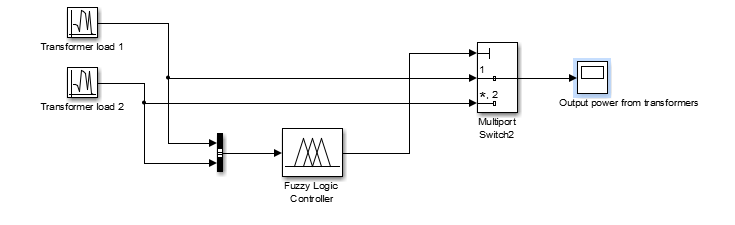


Figure S6. Overall protection systems for overloading protection/interface

Supplement: Supplementary file 6 — Additional file 6: Figure S6. Overall protection systems for overloading protection/interface. [file 13104_2019_4880_MOESM6_ESM.docx]
